# Diagnostic Delay in Gaucher Disease and the Role of Gastroenterology in the Diagnostic Journey: A Real-World Cohort Study

**DOI:** 10.3390/jcm15197472

**Published:** 2026-09-25

**Authors:** Bengi Ozturk, Muhammet Furkan Cakmak, Abdurrahman Ozkan, Ibrahim Nahit Sendur, Onur Keskin, Taylan Kav

**Affiliations:** Department of Gastroenterology, Faculty of Medicine, Hacettepe University, Ankara 06230, Türkiye

**Keywords:** Gaucher disease, diagnostic delay, gastroenterology

## Abstract

**Background/Objectives:** Gaucher disease (GD) is a rare lysosomal storage disorder with heterogeneous and often nonspecific clinical manifestations. Despite increasing awareness of Gaucher disease (GD), diagnostic delay remains a clinical challenge because of its heterogeneous and nonspecific presentation. This study evaluated the diagnostic journey of patients with GD, with particular emphasis on diagnostic delay, healthcare specialties encountered before diagnosis, and the involvement of gastroenterology in the diagnostic pathway. **Materials and Methods:** Patients with GD followed at the Hacettepe University Department of Gastroenterology between January 2014 and December 2024 were included. Demographic and clinical characteristics, age at symptom onset and diagnosis, diagnosis delay, medical specialties consulted before diagnosis, diagnostic methods, GD subtype, and treatment data were obtained from hospital records. Continuous variables were summarized as medians, minimum-maximum values, or interquartile ranges as appropriate, while categorical variables were summarized as frequencies and percentages. **Results:** Of the 34 patients, 22 (64.7%) were female, and 12 (35.3%) were male. The median age at diagnosis was 17.5 years [minimum(min)–maximum(max), 1–69], and the median age at symptom onset was 9 years (min–max, 1–44). Diagnostic-delay data were available for 21 patients. The median time from symptom onset to diagnosis was 1 year (range, 0–19); 11/21 patients (52.4%) were diagnosed within 1 year, whereas 5/21 (23.8%) experienced a diagnostic delay of ≥5 years. Among patients with available data on the diagnosing specialty, 42.9% were diagnosed in pediatric gastroenterology and 28.6% in adult gastroenterology. Among 23 patients with available prediagnostic referral data, 8 (34.8%) had consulted two or more different medical specialties before diagnosis. **Conclusions:** Although more than half of the patients with available diagnostic-delay data were diagnosed within 1 year of symptom onset, nearly one-quarter experienced delays of ≥5 years. Gastroenterology services represented a frequent point of diagnosis in this referral-center cohort, although this observation should be interpreted in light of the gastroenterology-based study setting and associated referral and selection bias. These findings should be interpreted cautiously given the small sample size and missing data. Greater awareness of GD in patients presenting with unexplained hepatosplenomegaly and/or cytopenias, together with timely use of disease-specific biochemical and molecular testing, may help shorten the diagnostic journey and reduce unnecessary diagnostic procedures.

## 1. Introduction

Gaucher disease (GD) is an autosomal recessive metabolic disorder caused by mutations in the GBA1 gene located on chromosome 1q21. It is characterized by a marked decrease in the activity of the lysosomal acid beta-glucocerebrosidase enzyme [1,2,3]. This enzyme deficiency leads to the progressive accumulation of lipid substrates such as glucosylceramide and its deacylated derivative, glucosylsphingosine (lyso-Gb1), which can easily diffuse into fluids and accumulate in the lysosomes of macrophages. These lipid-laden macrophages, called “Gaucher cells,” infiltrate the reticuloendothelial system, primarily the spleen, liver, and bone marrow [1], causing progressive multiorgan dysfunction [1,3,4]. Hepatosplenomegaly, anemia and thrombocytopenia, chronic fatigue, bleeding tendencies, neurological disorders, osteopenia, osteonecrosis, and pathological fractures may be observed [5,6,7]. The rarity of the disease and the nonspecific nature of its clinical symptoms make the diagnostic process difficult [8,9]. The time between the onset of first symptoms and reaching a definitive diagnosis often results in diagnostic delays lasting years, and in some cases even decades [10,11,12]. One of the most important reasons for this delay is the insufficient awareness of the disease among clinicians. International studies have shown that only 20% of hematologists and oncologists consider GD as a preliminary diagnosis in a patient presenting with the classic symptom cluster of anemia, thrombocytopenia, hepatosplenomegaly, and bone pain; whereas physicians primarily suspect hematological malignancies such as leukemia, lymphoma, or multiple myeloma [13,14]. Consequently, the diagnostic pathway of GD may be prolonged, particularly when its multisystem manifestations are evaluated separately by different specialties.

Patients with delayed diagnosis, especially in the presence of thrombocytopenia, may be subjected to unnecessary and invasive procedures such as liver biopsy or bone marrow biopsy, or may undergo unnecessary surgical splenectomies due to splenomegaly [9,15]. Most importantly, diagnostic delays can lead to irreversible skeletal damage such as osteonecrosis, permanent joint destruction, vertebral collapse, and pathological fractures [9].

Today, GD has highly effective specific treatments, such as enzyme replacement therapy (ERT) and substrate reduction therapy (SRT), that can reverse systemic symptoms and prevent further deterioration. These treatments must be initiated early, before clinical complications and permanent tissue damage develop [5,16].

Although GD has traditionally been strongly associated with hematology and metabolic disease practice, gastroenterologists may also encounter undiagnosed patients because hepatomegaly, splenomegaly, and abdominal manifestations are common features of the disease [6]. In particular, the combination of otherwise unexplained hepatosplenomegaly and cytopenias may provide an important opportunity for earlier recognition. This is particularly relevant to gastroenterology practice, as liver and splenic involvement may prompt referral for the evaluation of unexplained organomegaly before GD is considered. A practical review specifically addressing hepatologists and gastroenterologists has emphasized the need to consider GD in the differential diagnosis of unexplained splenomegaly and hepatosplenic abnormalities [6]. More recently, Aragon et al. identified gastroenterology, together with internal medicine, among the specialties in which greater awareness of GD may help address ongoing diagnostic delays [8].

Although previous studies have characterized diagnostic delays, physician awareness, and referrals across multiple specialties in GD, the specific role of gastroenterology in the diagnostic pathway remains less well characterized [8,10,13,14]. However, real-world data specifically examining the contribution of gastroenterology to establishing the diagnosis are limited. In particular, it remains unclear whether gastroenterology primarily serves as a referral and follow-up setting for patients with an already established or suspected diagnosis or may also represent a point of initial recognition and diagnosis in patients without prior suspicion of GD.

Therefore, this study aimed to characterize the diagnostic journey of patients with GD followed at a tertiary gastroenterology referral center, focusing on the time from symptom onset to definitive diagnosis, the medical specialties consulted before diagnosis, the diagnostic procedures performed, and the specialty in which the diagnosis was established. We also sought to specifically describe the involvement of gastroenterology in the diagnostic pathway, including the identification of patients in whom GD was diagnosed during gastroenterological evaluation without a prior suspicion or diagnosis of GD, and to identify potential opportunities for earlier disease recognition in gastroenterology practice.

## 2. Materials and Methods

### 2.1. Study Population

This retrospective, single-center observational study included patients with Gaucher disease (GD) followed at the Department of Gastroenterology, Hacettepe University Faculty of Medicine, between January 2014 and December 2024. Patients were eligible for inclusion if they were aged ≥18 years and were being followed in the adult gastroenterology department with a diagnosis of GD. Patients younger than 18 years of age and those who were not followed in the adult gastroenterology department were excluded. Patients diagnosed during childhood in pediatric departments were included if they had subsequently transitioned to follow-up in the adult gastroenterology department after the age of 18 years. Pediatric departments were not separate recruitment sites for this study.

For this retrospective study, GD diagnosis was determined from the diagnostic information documented in the medical records. Diagnostic evidence included reduced glucocerebrosidase enzyme activity, GBA1 genetic analysis, and/or bone marrow findings consistent with GD. Enzyme and genetic testing were not available for all patients, and therefore confirmation by both methods was not required for inclusion.

The study was approved by the Hacettepe University Faculty of Medicine Ethics Committee (approval no. 2026/10-44 and date: 12 May 2026) and was conducted in accordance with the Declaration of Helsinki.

### 2.2. Data Collection and Study Definitions

Patients’ demographic and clinical data were retrospectively extracted from the hospital information management system and electronic medical records. The collected variables included sex, year of birth, age at diagnosis, education level, region of birth, GD subtype, method of diagnosis, age at first symptom onset, type of initial symptom, time from symptom onset to definitive diagnosis, disease-specific treatment, the clinical department in which the diagnosis was established, and the number of different medical specialties consulted before diagnosis. Available clinical notes, laboratory results, diagnostic test reports, and consultation records were reviewed to verify the extracted information. When information was unclear or inconsistent across records, the available source documents were re-reviewed before the variable was recorded. Data that could not be established from the available medical records were classified as missing and were not inferred or imputed.

Diagnostic delay was defined as the interval between the documented onset of the first symptom or clinical finding attributable to GD and the date of definitive diagnosis. When the exact date was unavailable, the earliest documented age or year of symptom onset recorded in the medical record was used. Patients without GD-related symptoms at the time of initial evaluation who were investigated because of incidentally detected organomegaly or screening prompted by a family history of GD were classified as asymptomatic at presentation. For descriptive analyses, diagnostic delay was categorized as ≤1 year, >1 to <5 years, and ≥5 years. These thresholds were selected to distinguish relatively early diagnosis from intermediate and prolonged diagnostic delay and were informed by previous GD studies in which delays exceeding 1 year and delays of ≥5 years have been used or specifically reported as clinically relevant measures of diagnostic delay [8,12]. For the purposes of the present study, a diagnostic delay of ≥5 years was considered prolonged diagnostic delay.

### 2.3. Statistical Analyses

Statistical analyses were performed using IBM SPSS Statistics, version 30.0. Continuous variables were summarized as medians with either minimum–maximum values or interquartile ranges (IQR), as appropriate, while categorical variables were presented as frequencies and percentages. No imputation was performed for missing data because missing values largely represented historical clinical information that could not be reliably reconstructed from the available medical records. Analyses were therefore conducted using available-case data, and the denominator for each variable was based on the number of patients with available information. Given the small sample size and descriptive nature of the study, no formal sensitivity analyses for missing data were performed.

## 3. Results

There were 34 patients followed up with a diagnosis of GD. 22 (64.7%) of the patients were female and 12 (35.3%) were male. The median age at diagnosis was 17.5 years (min–max 1–69). The median age at symptom onset was 9 years (min–max: 1–44). Patients’ characteristics are shown in Table 1. Information regarding symptomatic status at presentation was available for 26 patients. Of these, 22/26 (84.6%) were symptomatic (Table 2). Four patients (15.4%) had no reported symptoms at diagnosis. Of these, one was diagnosed through family screening after the diagnosis of GD in a sibling, while two were diagnosed during the evaluation of hepatosplenomegaly. Detailed information on the diagnostic presentation was unavailable for one patient. Symptoms varied. Among the 22 symptomatic patients, gastrointestinal symptoms, including abdominal pain, nausea, and vomiting, were the most frequently recorded initial symptoms (7/22, 31.8%). Data on the interval between first symptom onset and definitive diagnosis were available for 21 patients. The median diagnostic delay was 1 year (range, 0–19 years) (Table 2).

Among these 21 patients, 11 (52.4%) had a diagnostic delay of ≤1 year, 5 (23.8%) had a delay of >1 to <5 years, and 5 (23.8%) had a delay of ≥5 years. The clinical characteristics of the five patients with prolonged diagnostic delay (≥5 years) were examined separately (Table 3). Three patients were male and two were female. Notably, symptom onset occurred before 5 years of age in four of the five patients (80%), whereas one patient developed symptoms at 31 years of age. Despite this similarity in early symptom onset, the initial clinical manifestations were heterogeneous, including gastrointestinal symptoms, fatigue, hepatosplenomegaly, hand tremor, and tic disorder. Information on the clinical department in which the diagnosis was established was available for 28 patients. Of these, 20 patients had already been diagnosed with GD by other specialties, including pediatric gastroenterology (*n* = 12, 42.9%), hematology (*n* = 5, 17.9%), pediatric hematology (*n* = 2, 7.1%), and pediatric metabolic diseases (*n* = 1, 3.6%), and were subsequently referred to the adult gastroenterology department for follow-up. In contrast, 8 patients (28.6%) were referred to adult gastroenterology without a prior suspicion or diagnosis of GD, and the diagnosis was established during their evaluation in the adult gastroenterology department. Data on the number of different medical specialties consulted before diagnosis were available for 23 patients. Prior to diagnosis, 15 (65.2%) patients had attended one medical specialty, 5 (21.7%) had attended two different medical specialties, 1 (4.3%) had attended three, and 2 (8.7%) had attended four. Information on diagnostic methods was available for 23 patients. A single diagnostic method was recorded in 12/23 patients (52.2%), two methods in 10/23 (43.5%), and three methods in 1/23 (4.3%) (Table 2). Regarding the diagnostic work-up, GBA1 genetic analysis was recorded in 12 patients, β-glucocerebrosidase enzyme activity testing in 12 patients, and bone marrow biopsy in 11 patients. The summary of the diagnostic journey of patients with GD in Figure 1. Regarding GD subtype, 31/34 patients (91.2%) had type 1 disease and 3/34 (8.8%) had type 3 disease; no patients with type 2 disease were identified. Regarding treatment, 32 (94.1%) patients received imiglucerase, while 1 (2.9%) received taliglucerase and 1 (2.9%) received miglustat (Table 1).

## 4. Discussion

Gaucher disease is one of the rare diseases where diagnostic delays persist due to the heterogeneous and often nonspecific nature of its clinical findings [8,9,10,12,13,14,17,18]. Our findings demonstrate that the diagnostic pathway of GD can vary considerably, even within a single tertiary referral cohort. Although the median diagnostic delay was relatively short, a subgroup of patients experienced prolonged delays. This may reflect the heterogeneous and multisystem nature of GD, which can lead patients to be evaluated by different medical specialties before the diagnosis is considered. Our findings are broadly consistent with previous studies showing substantial diagnostic delays in GD, although some differences were observed. In our cohort, the median diagnostic delay was 1 year, and 23.8% of patients with available data experienced a delay of ≥5 years. Thomas et al., in a cohort of 86 patients, reported a median diagnostic delay of 2 years, with 19% experiencing delays of ≥5 years [12]. Mehta et al. reported that approximately one-sixth of patients waited ≥7 years between the initial physician visit and definitive diagnosis [14]. More recently, Aragon et al., in an international survey including 142 respondents from 40 countries, reported that 58% waited more than 1 year for diagnosis and 46% consulted three or more specialists during their diagnostic journey [8]. In contrast, 65.2% of patients with available data in our cohort had consulted only one specialty before diagnosis. These differences may reflect variation in study populations, healthcare settings, and referral pathways.

Patients with prolonged diagnostic delay were also evaluated separately. Among the five patients with a diagnostic delay of ≥5 years, four had symptom onset before the age of 5 years. This finding suggests that early symptom onset may not always result in early diagnosis of GD. The initial clinical manifestations varied among these patients and included gastrointestinal symptoms, fatigue, hepatosplenomegaly, and neurological symptoms. The variability in initial presentation may make early recognition of GD more difficult and may contribute to diagnostic delay. However, given the small number of patients in this subgroup, these findings should be interpreted cautiously and considered descriptive.

The heterogeneous and multisystemic clinical features of GD can be one of the main reasons for delays in diagnosis. The disease can affect different organs and systems, primarily with gastrointestinal, hematological, skeletal, and neurological findings, and initial symptoms are often not specific to the disease [5,9,10,14]. The involvement of multiple medical specialties before diagnosis may reflect the multisystem and nonspecific presentation of GD. Patients may be evaluated by different specialties according to their initial clinical findings, which may make recognition of GD more difficult. Similar complex diagnostic pathways involving multiple physicians and specialties have also been reported in previous studies [8,14]. Greater awareness of GD among physicians from different specialties may therefore facilitate its earlier consideration in the diagnostic process.

The pattern of specialty involvement in our cohort differed from that reported in previous studies. Thomas et al. reported that 75% of patients were diagnosed by hematologists [12]. In our cohort, 15 of the 28 patients with available data (53.6%) had been diagnosed in pediatric departments before transitioning to adult care, including 12 in pediatric gastroenterology. Therefore, the specialty distribution should be interpreted in the context of our adult gastroenterology-based cohort. Notably, eight patients were diagnosed during adult gastroenterology evaluation without a prior suspicion or diagnosis of GD. This finding suggests that adult gastroenterologists may contribute to the initial recognition of GD, particularly in patients with unexplained hepatosplenic findings.

The coexistence of splenomegaly and thrombocytopenia is a particularly important clinical clue for the early diagnosis of Gaucher disease. A screening study conducted by Huang et al. in high-risk individuals with splenomegaly and/or thrombocytopenia demonstrated that Gaucher patients can be identified through targeted screening strategies [19]. Similarly, Motta et al. developed a clinical approach to predict the likelihood of Gaucher disease in individuals with splenomegaly and thrombocytopenia [20]. The study by Mehta et al. and the subsequently developed scoring system also support the importance of splenomegaly, thrombocytopenia, and related clinical findings in early diagnostic algorithms [9,21]. These data suggest that the coexistence of unexplained splenomegaly and cytopenias should be considered an important clinical warning sign for Gaucher disease, particularly in gastroenterology and hematology practice. These findings have practical implications for the earlier recognition of GD. Gastroenterologists should consider GD in patients with unexplained splenomegaly or hepatosplenomegaly, particularly when accompanied by cytopenias or other compatible clinical findings. Early consideration of GD may allow timely use of disease-specific biochemical and genetic testing and may reduce unnecessary invasive investigations. Increasing awareness among gastroenterologists, hematologists, internists, and pediatricians, together with incorporating GD into diagnostic approaches for unexplained splenomegaly and cytopenias, may improve diagnostic efficiency. Multidisciplinary collaboration may also facilitate earlier diagnosis in patients with complex or multisystem presentations.

Bone marrow examination in some patients may reflect the hematological presentation of GD, which can lead to evaluation for hematological malignancies or bone marrow disorders. Previous studies have reported that Gaucher patients may undergo bone marrow biopsy and other invasive diagnostic procedures before definitive diagnosis [9,13,15]. In patients with unexplained splenomegaly and cytopenias, earlier consideration of GD may facilitate the timely use of disease-specific biochemical and molecular tests and potentially reduce the need for invasive diagnostic procedures. The coexistence of anemia, thrombocytopenia, and splenomegaly may particularly lead to investigations for hematological malignancies or primary bone marrow diseases [9,13]. However, in current diagnostic approaches, demonstrating a decrease in β-glucocerebrosidase enzyme activity is one of the fundamental diagnostic methods for confirming Gaucher disease, and molecular analysis of the GBA1 gene plays an important role in confirming the diagnosis and determining the genotype [3]. Greater awareness of GD among clinicians evaluating compatible clinical presentations may therefore facilitate earlier use of disease-specific biochemical testing and potentially reduce the need for invasive investigations in selected patients.

In our study, 91.2% of patients had type 1 Gaucher disease, 8.8% had type 3, and no patients with type 2 disease were found. The predominance of type 1 disease is consistent with the known clinical distribution of Gaucher disease [2,22,23]. However, the fact that the age at diagnosis varies between 1 and 69 years is important in demonstrating the wide heterogeneity in the clinical presentation of the disease.

This study has several limitations. First, its retrospective, single-center design and relatively small sample size limit the generalizability of the findings. Second, historical data regarding symptom onset, diagnostic delay, the number of medical specialties consulted before diagnosis, and diagnostic methods were not available for all patients. In particular, diagnostic-delay data were available for only 21 of the 34 patients. The use of available-case analysis in the presence of substantial missing data may have introduced bias if patients with available information differed systematically from those with missing information and may have reduced the precision and robustness of the corresponding estimates. Given the retrospective nature and small sample size of the study, missing historical information could not be reliably reconstructed, and imputation or formal sensitivity analyses were not performed. Therefore, findings based on variables with incomplete data should be considered descriptive and interpreted cautiously. The potential effects of educational, socioeconomic, and geographic factors on diagnostic delay could not be adequately assessed because of incomplete sociodemographic data. Educational level was available for 17 patients and region of birth for 21 patients. Moreover, region of birth may not accurately reflect current access to healthcare, and occupational data alone were insufficient to characterize socioeconomic status. Therefore, these variables were reported descriptively but were not analyzed in relation to diagnostic delay. Larger prospective studies with more comprehensive sociodemographic data are needed to evaluate their potential influence on diagnostic delay. The retrospective determination of symptom onset is also susceptible to incomplete documentation and recall bias. Third, the small sample size and missing data precluded robust evaluation of factors independently associated with prolonged diagnostic delay. Fourth, specific alternative or provisional diagnoses considered before GD diagnosis were not systematically documented, limiting our ability to identify the most common causes of diagnostic misdirection. Finally, because the study population consisted of patients followed at a tertiary gastroenterology referral center, referral and selection bias are likely. Patients with gastrointestinal or hepatosplenic manifestations may be overrepresented, and the observed frequency of diagnosis by gastroenterology services may therefore be higher than that in the broader GD population.

## 5. Conclusions

In this retrospective cohort of patients with GD followed at a tertiary gastroenterology referral center, the median diagnostic delay was 1 year; however, nearly one-quarter of patients with available data experienced a delay of ≥5 years, and approximately one-third consulted two or more different medical specialties before receiving a definitive diagnosis. Gastroenterology services represented a frequent point of diagnosis in this cohort, although this finding should be interpreted with caution because of the selection and referral bias inherent to the gastroenterology-based study setting. The small sample size and substantial amount of missing data further limit the generalizability of these findings to the broader GD population. Greater awareness of GD among clinicians evaluating compatible clinical presentations, together with timely use of disease-specific biochemical and molecular testing, may facilitate earlier diagnosis and potentially reduce prolonged diagnostic pathways and invasive diagnostic procedures in selected patients.

## Figures and Tables

**Figure 1:**
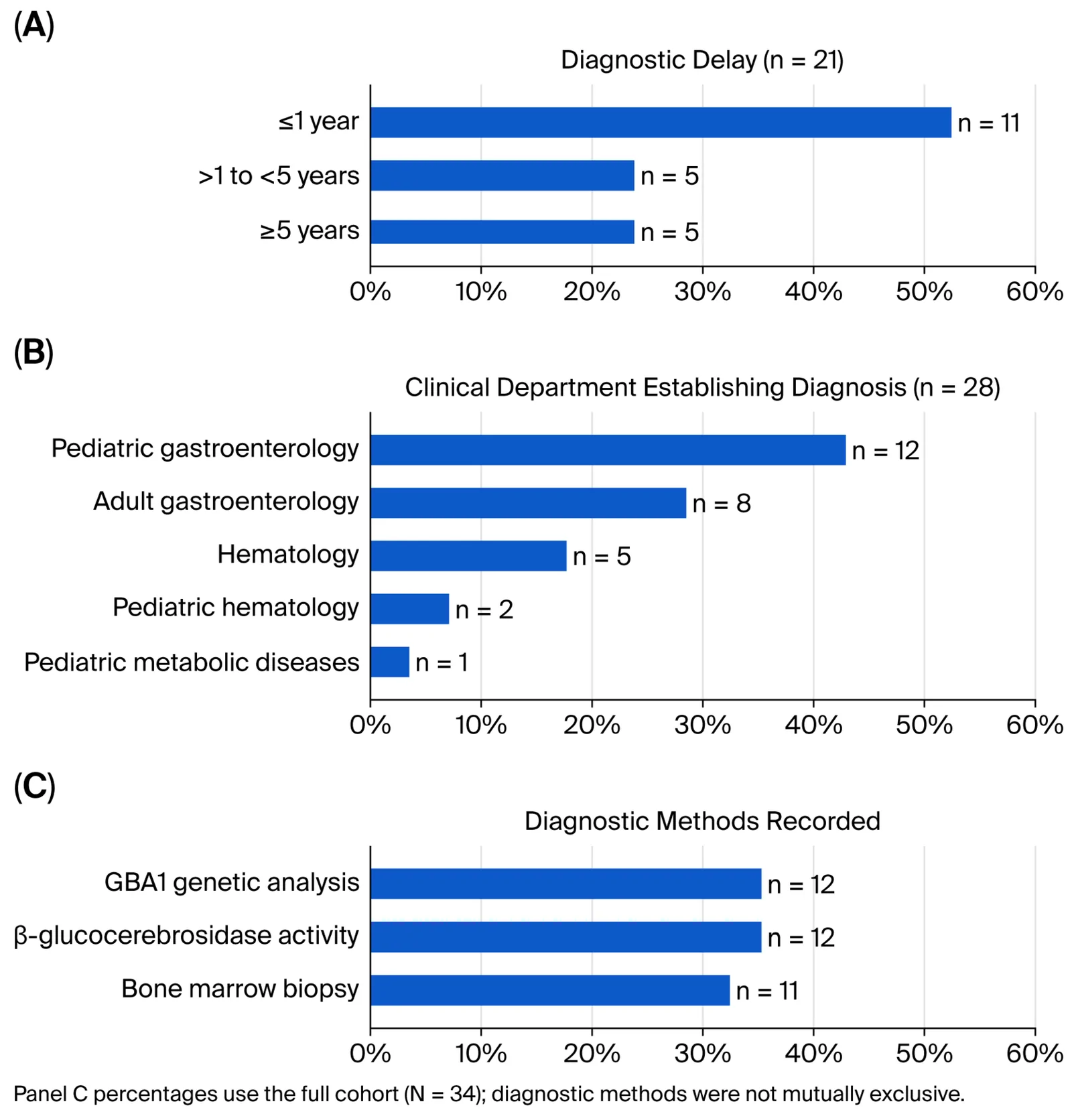
Summary of the diagnostic journey of patients with Gaucher disease. (**A**) Diagnostic delay from symptom onset to diagnosis. (**B**) Clinical department in which the diagnosis of Gaucher disease was established. (**C**) Diagnostic methods recorded, including GBA1 genetic analysis, β-glucocerebrosidase activity measurement, and bone marrow biopsy.

**Table 1:** Demographic and Clinical Characteristics of the Study Population.

Characteristic	Value
Total number of patients	34
Female sex, *n* (%)	22 (64.7)
Male sex, *n* (%)	12 (35.3)
Age at diagnosis, years, median (range)	17.5 (1–69)
Age at first symptom onset, years, median (range)	9 (1–44)
Education Level (*N* = 17)	
Primary school, *n* (%)	2 (11.8)
Middle school, *n*(%)	2 (11.8)
High school, *n* (%)	5 (29.4)
University graduate, *n* (%)	8 (47.1)
Region of birth (*N* = 21)	
Southeastern Anatolia, *n* (%)	5 (23.8)
Eastern Anatolia, *n* (%)	4 (19)
Marmara, *n* (%)	1 (4.8)
Black Sea, *n* (%)	4 (19.0)
Central Anatolia, *n* (%)	5 (23.8)
Mediterranean, *n* (%)	1 (4.8)
Aegean, *n* (%)	1 (4.8)
GD subtype	
Type 1, *n* (%)	31 (91.2)
Type 2, *n* (%)	0
Type 3, *n* (%)	3 (8.8)
Disease-specific treatment	
Imiglucerase	32 (94.1)
Taliglucerase	1 (2.9)
Miglustat	1 (2.9)

Data are presented as *n* (%) unless otherwise indicated. GD, Gaucher disease.

**Table 2:** Diagnostic Journey of Patients with Gaucher Disease.

Characteristic	Value
Symptomatic status at presentation (*n* = 26)	
Symptomatic, *n*/*N* (%)	22/26 (84.6)
Asymptomatic, *n*/*N* (%)	4/26 (15.4)
Diagnostic delay (*n* = 21)	
Diagnostic delay, years, median (range)	1 (0–19)
≤1 year, *n*/*N* (%)	11/21 (52.4)
>1 to <5 years, *n*/*N* (%)	5/21 (23.8)
≥5 years, *n*/*N* (%)	5/21 (23.8)
Number of different medical specialties consulted before diagnosis (*n* = 23)	
1 specialty, *n*/*N* (%)	15/23 (65.2)
2 specialty, *n*/*N* (%)	5/23 (21.7)
3 specialty, *n*/*N* (%)	1/23 (4.3)
4 specialty, *n*/*N* (%)	2/23 (8.7)
Clinical department establishing the diagnosis (*n* = 28)	
Pediatric gastroenterology, *n*/*N* (%)	12/28 (42.9)
Adult gastroenterology, *n*/*N* (%)	8/28 (28.6)
Hematology, *n*/*N* (%)	5/28 (17.9)
Pediatric hematology, *n*/*N* (%)	2/28 (7.1)
Pediatric metabolic diseases, *n*/*N* (%)	1/28 (3.6)
Number of diagnostic methods recorded (*n* = 23)	
One method, *n*/*N* (%)	12/23 (52.2)
Two methods, *n*/*N* (%)	10/23 (43.5)
Three methods, *n*/*N* (%)	1/23 (4.3)

Data are presented as *n*/*N* (%) unless otherwise indicated. Percentages were calculated using the number of patients with available data for each variable as the denominator.

**Table 3:** Clinical Characteristics of Patients with Prolonged Diagnostic Delay (≥5 Years).

Patient	Sex	Age at Diagnosis (Years)	Diagnostic Methods	Age at Symptom Onset (Years)	Symptoms	Department Establishing the Diagnosis
1	F	17	Genetic mutation, enzyme level	2	Gastrointestinal symptom	Pediatric gastroenterology
2	M	46	Genetic mutation, bone marrow biopsy	31	Fatigue	Hematology
3	M	10		2	Fatigue, gastrointestinal symptom, HSM	Pediatric gastroenterology
4	F	18	Enzyme level	4	Tremor	Pediatric gastroenterology
5	M	20	Genetic mutation, enzyme level	1	Tic disorder	Adult gastroenterology

Abbreviations: F: female, M: male, HSM: hepatosplenomegaly.

## Data Availability

Data is unavailable due to privacy or ethical restrictions.
